# Editorial: Hypoxia and angiogenesis in cancer

**DOI:** 10.3389/fphar.2024.1448204

**Published:** 2024-08-13

**Authors:** Mohammad Malekan, Nicholas Denko, Lorenzo Mortara, Appu Rathinavelu, Mohammad Mahdi Parvizi, Umamaheswari Natarajan

**Affiliations:** ^1^ Student Research Committee, School of Medicine, Mazandaran University of Medical Sciences, Sari, Iran; ^2^ The Ohio State University Wexner Medical Center and OSU Comprehensive Cancer Center, Columbus, OH, United States; ^3^ Laboratory of Immunology and General Pathology, Department of Biotechnology and Life Sciences, University of Insubria, Varese, Italy; ^4^ Unit of Molecular Pathology, Biochemistry and Immunology, IRCCS MultiMedica, Milan, Italy; ^5^ Rumbaugh-Goodwin Institute for Cancer Research, Nova Southeastern University, Fort Lauderdale, FL, United States; ^6^ Molecular Dermatology Research Center, Shiraz University of Medical Sciences, Shiraz, Iran

**Keywords:** angiogenesis, hypoxia, cancer, signaling pathways, HIF, VEGF, VEGFR

Progress in understanding the mechanisms involved in cancer has led to in-depth cancer knowledge and novel treatment approaches. Some of these novel approaches have revolutionized cancer treatment, leading to a substantial improvement in patient overall survival. Nonetheless, cancer patients still pose a significant challenge to global health, and its worldwide burden continues to increase (Yuan et al., 2024).

The processes of angiogenesis and hypoxia are essential in tumorigenesis and closely linked to almost all forms of cancer. Angiogenesis, which involves the creation of new blood vessels, is a critical mechanism that facilitates tumor development by providing nourishment and oxygen (Malekan et al., 2024). Conversely, hypoxia, characterized by low oxygen levels, frequently occurs within tumors as a result of rapid cell growth surpassing the oxygen supply (Malekan et al., 2021). Understanding the knowledge behind hypoxia and angiogenesis in cancer and their relationship is vital since they exert the activation of survival pathways, promoting cancer cell survival and resistance to treatments. In addition, these two processes mediate various factors and signaling pathways. These factors include hypoxia-inducible factors (HIFs), vascular endothelial growth factors (VEGFs), and their receptors (VEGFRs). They work with signaling pathways such as MAPK/ERK, PKC, PLC-γ, FAK, p38, and PI3K (Heer et al., 2020). In this Research Topic, we have included four original research articles and one review paper on the topic of “*Hypoxia and angiogenesis in cancer*.” In the following paragraphs, we will briefly go through these articles.

Park et al. present an encouraging therapeutic agent for cancer treatment. Their study introduces CU05-1189, a new small molecule that specifically targets the pleckstrin homology domain of PDK1. This molecule significantly inhibits VEGF-induced proliferation, migration, invasion, and tube formation in human umbilical vein endothelial cells. Upon VEGF stimulation, the Akt signaling pathway is particularly suppressed by CU05-1189. When CU05-1189 is administered orally, it leads to a decrease in tumor microvessel density and growth in a xenograft mouse model. These findings indicate that CU05-1189 could potentially serve as an effective new antiangiogenic agent in cancer therapy (Park et al.).

Han et al. present a detailed study on the role of hypoxia in osteosarcoma, a prevalent malignant bone tumor in both children and adolescents. The researchers established a reliable risk prognostic model based on 200 hypoxia-linked genes. Their comprehensive analysis resulted in a reliable risk prognostic model capable of accurately forecasting patient outcomes. They identified two molecular subgroups with significantly different survival rates and formulated a risk model based on 12 genes. The study discovered that high-risk patients had lower Tfh cell infiltration and a lower stromal score. They also found that KCNJ3 could be a significant prognostic indicator for the progression of osteosarcoma (Han et al.).

Qin et al. explore the role of Tanshinone IIA (Tan IIA) in liver cancer treatment. The study reveals that Tan IIA, known for enhancing microcirculation, does not inhibit tumor growth directly but significantly boosts the inhibitory effect of Sorafenib on liver cancer. It assists in preserving the normal structure of blood vessels, prevents liver cancer cells from attracting vascular endothelial cells, and mitigates the hypoxia in the tumor microenvironment. This is accomplished by diminishing the expression of HIF-1α and HIF-2α through the PI3K-AKT signaling pathway. The findings provide a theoretical basis for the clinical transformation and usage of Tan IIA (Qin et al.).

Ou et al. investigate how hypoxia contributes to the immune evasion of pancreatic cancer. The study reveals that hypoxic conditions in the tumor microenvironment impair the functionality of natural killer (NK) cells, contributing to tumor immune escape. The research focuses on the impact of miR-1275/AXIN2 on NK cells. The findings suggest that hypoxia considerably lowers miR-1275 expression, which in turn diminishes the killing capacity of NK cells. The increase in miR-1275 expression boosts the cytotoxicity of NK cells, and miR-1275 is observed to bind to and suppress AXIN2 expression. This study emphasizes the pivotal role of the miR-1275/AXIN2 pathway in the immune evasion of pancreatic cancer caused by hypoxia (Ou et al.).

Wang et al. provide a comprehensive overview of the progress made in anti-angiogenic therapy. The paper underscores the importance of VEGF and VEGFR as key targets in this therapeutic approach. Over the years, a variety of drugs have been developed that target the VEGF/VEGFR axis, offering potential treatments for a range of cancers and retinopathies. These drugs, each with their unique molecular structures and properties, inhibit the VEGF/VEGFR interaction, the activity of VEGFR tyrosine kinase, or VEGFR downstream signaling. The article not only reviews these drugs but also discusses their mechanisms of action, clinical benefits, and the challenges that future anti-angiogenic drugs need to address (Wang et al.).

Emerging research has indeed begun to unravel the complex mechanisms underlying angiogenesis and hypoxia in cancer. This deep understanding has paved the way for the development of novel therapeutic approaches, which have shown promise in improving survival rates. There are multiple FDA-approved anti-angiogenic agents such as bevacizumab, ramucirumab, aflibercept, sorafenib, sunitinib, pazopanib, regorafenib, and cabozantinib which have shown efficacy in specific cancers. In spite of various FDA-approved anti-angiogenic drugs, few agents have been developed and received approval to target hypoxia in cancer. Belzutifan (MK-6482) is a hypoxia-inducible factor-2 alpha (HIF-2α) inhibitor. It received FDA approval for the treatment of von Hippel-Lindau (VHL) disease-associated renal cell carcinoma (RCC) in adults. By inhibiting HIF-2α, belzutifan helps suppress tumor growth and progression in patients. Therapies designed to target specific molecular pathways involved in angiogenesis and hypoxia have the potential to be more effective and less toxic than traditional cancer treatments. Additionally, these drugs can make tumors more susceptible to other treatments such as radiation and chemotherapy.

Furthermore, the use of advanced technologies, such as next-generation sequencing and bioinformatics, are enabling researchers to achieve a more comprehensive understanding of the genetic and molecular aspects of tumors. This may lead to the discovery of new therapeutic targets and the creation of personalized treatment approaches based on the specific characteristics of each patient’s tumor.

In conclusion, despite all these advances, there are still many challenges to overcome regarding cancer treatment. For example, tumors may become resistant to targeted therapies, and the efficacy of these treatments can differ significantly among patients. Future research should prioritize addressing these challenges and investigating novel approaches to improve the efficiency of treatments. Studying hypoxia and angiogenesis in cancer has the potential to help develop new targeted therapies against different forms of cancers. Continued research and innovation offer hope for creating more effective treatments, ultimately improving the survival and quality of life of cancer patients.
